# Blood‐derived DNA methylation predictors of mortality discriminate tumor and healthy tissue in multiple organs

**DOI:** 10.1002/1878-0261.12738

**Published:** 2020-06-22

**Authors:** Yan Zhang, Melanie Bewerunge‐Hudler, Matthias Schick, Barbara Burwinkel, Esther Herpel, Michael Hoffmeister, Hermann Brenner

**Affiliations:** ^1^ Division of Clinical Epidemiology and Aging Research German Cancer Research Center (DKFZ) Heidelberg Germany; ^2^ German Cancer Consortium (DKTK) German Cancer Research Center (DKFZ) Heidelberg Germany; ^3^ Genomics and Proteomics Core Facilities Microarray Unit German Cancer Research Center (DKFZ) Heidelberg Germany; ^4^ Molecular Epidemiology German Cancer Research Center Heidelberg Germany; ^5^ Molecular Biology of Breast Cancer Department of Obstetrics and Gynecology University of Heidelberg Heidelberg Germany; ^6^ Department of General Pathology Institute of Pathology University of Heidelberg Heidelberg Germany; ^7^ NCT Tissue Bank National Center for Tumor Diseases (NCT) Heidelberg Germany; ^8^ Division of Preventive Oncology German Cancer Research Center (DKFZ) and National Center for Tumor Diseases (NCT) Heidelberg Germany

**Keywords:** blood and tissues, cancer, DNA methylation, DNAmPhenoAge, mortality risk score

## Abstract

Evidence has shown that certain methylation markers derived from blood can mirror corresponding methylation signatures in internal tissues. In the current study, we aimed to investigate two strong epigenetic predictors for life span, derived from blood DNA methylation data, in tissue samples of solid cancer patients. Using data from the Cancer Genome Atlas (TCGA) and the German DACHS study, we compared a mortality risk score (MRscore) and DNAmPhenoAge in paired tumor and adjacent normal tissue samples of patients with lung (*N* = 69), colorectal (*n* = 299), breast (*n* = 90), head/neck (*n* = 50), prostate (*n* = 50), and liver (*n* = 50) cancer. To explore the concordance across tissue and blood, we additionally assessed the two markers in blood samples of colorectal cancer (CRC) cases and matched controls (*n* = 93) in the DACHS+ study. The MRscore was significantly elevated in tumor tissues compared to normal tissues of all cancers except prostate cancer, for which an opposite pattern was observed. DNAmPhenoAge was consistently higher in all tumor tissues. The MRscore discriminated lung, colorectal, and prostate tumor tissues from normal tissues with very high accuracy [AUCs of 0.87, 0.99 (TCGA) /0.94 (DACHS), and 0.92, respectively]. DNAmPhenoAge accurately discriminated five types of tumor tissues from normal tissues (except prostate cancer), with AUCs of 0.82–0.93. The MRscore was also significantly higher in blood samples of CRC cases than in controls, with areas under the curve (AUC) of 0.74, whereas DNAmPhenoAge did not distinguish cases from controls, with AUC of 0.54. This study provides compelling evidence that blood‐derived DNAm markers could reflect methylation changes in less accessible tissues. Further research should explore the potential use of these findings for cancer diagnosis and early detection.

## Graphical abstract

In this study, methylation predictors of mortality, derived from blood DNA methylation analysis, were found to be remarkably elevated in tumor tissue, when compared to adjacent normal tissue of multiple organs. In patients with colorectal cancer, the mortality methylation predictor pattern was consistent across tumor tissue and peripheral blood. Thus, blood‐derived methylation markers could mirror tumorigenesis‐related methylation changes in less accessible tissues.

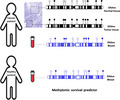

**Abbreviations**
AUCs: areas under the curve
BC: breast cancer
CI: confidence interval
CRC: colorectal cancer
CRP: C‐reactive protein
DNAm: deoxyribonucleic acid methylation
EWAS: epigenome‐wide association study
LC: lung cancer
MRscore: mortality risk score
ROC curves: receiver operating characteristic curves
TILs: tumor‐infiltrating leukocytes

## 1 Introduction

DNA methylation (DNAm), one of the key epigenetic processes, plays a critical role in tissue and cellular differentiation such that different tissues are characterized by distinct DNAm patterns [1,2]. Studies, however, via either direct comparing DNAm profiles across tissues [2,3] or exploring phenotype‐related DNAm alterations across tissues (such as adipose or brain tissues vs. blood, and buccal cells vs. blood), have shown concordance or correlation of a subset of methylation signatures between tissues [4, 5, 6, 7, 8], suggesting the potential of blood DNAm as a surrogate measure of methylation at less accessible internal tissues. In the field of aging research, Teschendorff *et al*. identified an age‐dependent signature based on 69 CpGs mapping to promoter of polycomb group proteins, which is common not only to multiple tissues including blood and epithelial tissues, but also to the process of carcinogenesis [9]. Horvath developed a 353‐CpG‐based multitissue age predictor, known as ‘Epigenetic clock’, which can accurately predict age across a variety of tissues (e.g., whole blood, blood mononuclear cells, colon, adipose, liver, and lung) [10]. These findings support the hypothesis that robust DNAm markers derived from blood are able to mirror those in solid tissues.

Recently, two strong predictors for life span (time‐to‐death due to all‐cause mortality) have been developed using blood DNAm profiles [11,12]. We first derived a 10‐CpG‐based mortality risk score (MRscore) from a whole blood epigenome‐wide association study (EWAS) of mortality [12]. The MRscore was shown to strongly predict all‐cause and cause‐specific mortality, and to strongly correlate with other well‐established aging indicators (such as telomere length, oxidative stress, frailty index, and the epigenetic clock), while outperforming these indicators in survival prediction [13, 14, 15]. The MRscore was first derived and validated in two large German cohorts, and its strong association with mortality has subsequently been confirmed in multiple large cohort studies from the United States, such as the Framingham Heart Study, the Women's Health Initiative, and the Normative Aging Study [11,16,17]. Later, Levine *et al*. [11] developed another survival predictor by regressing a phenotypic measure of mortality risk on 513 CpGs, that is, DNAmPhenoAge, which showed an association with mortality as strongly as that for the MRscore. Although it was also developed based on whole blood samples, the DNAmPhenoAge correlated to various extents with chronological age in multiple tissues (*r* = 0.47–0.92). A question of potential high clinical interest is to what extent methylation changes identified in blood to be predictive of major health outcomes would reflect methylation changes on tissue levels in the pathogenesis of major diseases, such as various cancers. The aim of the current study was to evaluate whether and to what extent the MRscore and DNAmPhenoAge would differ between cancer tissue and adjacent normal tissue for six common cancers, including lung, colorectal, breast, head/neck, prostate, and liver cancer. In parallel, we comparatively evaluated the epigenetic clock‐derived age acceleration, that is, DNAmAge acceleration, a well‐studied epigenetic marker derived from multitissue analysis, which has been shown to correlate with a wide spectrum of health conditions [18, 19, 20]. To assess the concordance from solid tissue to peripheral blood, we additionally analyzed the three markers in blood samples of colorectal cancer (CRC) cases and matched controls.

## 2 Materials and methods

### 2.1 Study population

The analysis of paired tumor‐normal tissues of six types of cancers was based on the Cancer Genome Atlas (TCGA) data, where DNAm data for tumor/adjacent normal tissues were extracted from patients with lung (*n* = 833/69), colorectal (*n* = 367/45), breast (*n* = 789/90), head/neck (*n* = 528/50), prostate (*n* = 498/50), and liver (*n* = 377/50) cancer. To preclude the impact of interindividual variation in DNAm, our analyses focused only on corresponding matched tumor and normal tissues from the same patients.

In addition, DNAm data of paired tumor and adjacent normal tissues of CRC patients were also drawn from the German DACHS study, an ongoing large population‐based case–control study on CRC. The DACHS study enrolls patients with histologically confirmed CRC from 22 hospitals in the Rhine–Neckar–Odenwald region in southwestern Germany [21,22]. For 254 patients diagnosed between 2003 and 2007, genome‐wide DNAm assessment in paired tumor‐normal tissues was available and included in the current study. The study was approved by the ethical committees of the University of Heidelberg and of the Medical Chambers of Baden‐Württemberg and Rhineland‐Palatinate. Written informed consent was obtained from each participant.

The analysis in blood samples was carried out in the German DACHS+ study, a satellite substudy to the DACHS study. In brief, the DACHS+ study recruited 819 CRC patients (age 55–75 years) referred by general practitioners or gastroenterologists for surgery to four hospitals in and around Heidelberg after diagnosis but before initiation of treatment between October 2006 and December 2014 [23]. Blood samples were obtained before surgery. Epigenome‐wide DNAm analysis using the Infinium HumanMethylation450K platform was conducted in blood samples of 93 randomly selected DACHS+ CRC cases and 94 age‐ and sex‐matched controls randomly selected from the Blitz Study, an ongoing epidemiological study recruiting participants (age 55–75 years) of screening colonoscopy in southwestern Germany [23]. The DACHS+ study was approved by the ethics committee of the University of Heidelberg.

Methodologies in the current study conformed to the standards set by the Declaration of Helsinki.

### 2.2 DNA methylation profiling and data preprocessing

For the TCGA samples, IDAT format files of the Infinium 450K methylation data were extracted from the TCGA website (https://portal.gdc.cancer.gov/legacy‐archive/search/f). DNAm of paired tissue samples of the DACHS and DACHS+ study was measured using the Infinium Methylation450K BeadChip (Illumina Inc., San Diego, CA, USA) at the Genomics and Proteomics Core Facility of the German Cancer Research Center, Heidelberg, Germany, according to the manufacturer's instructions. Details of DNA isolation from the tissue samples were described in a previous study [22]. All methylation data were preprocessed following the CPACOR pipeline [24]. Probes with detection *P*‐value > 0.01 and missing values > 5% were removed. Quantile normalization was applied following separating the probe type into six categories, based on probe type and color channel, using the ‘limma’ R package included in the Bioconductor [25]. Methylation beta values of the 10 CpGs and 513 CpGs, respectively, included in the MRscore and DNAmPhenoAge calculation were extracted. Horvath’s DNAmAge was calculated using the online tool available at https://dnamage.genetics.ucla.edu/.

### 2.3 Statistical analysis

The MRscore was computed in a continuous form as in our previous study [12], that is, as the sum of weighted methylation beta values of 10 CpGs: cg01612140*(−0.38253) + cg05575921*(−0.92224) + cg06126421*(−1.70129) + cg08362785*(2.71749) + cg10321156*(−0.02073) + cg14975410* (−0.04156) + cg19572487*(−0.28069) + cg23665802*(−0.89440) + cg24704287*(−2.98637) + cg25983901*(−1.80325). The weights were taken from the original study [12]. DNAmPhenoAge was calculated according to the formula reported by Levine *et al*. [11]. The DNAmAge acceleration was calculated as residuals of Horvath’s DNAmAge regressed on chronological age.

The levels of MRscore, DNAmPhenoAge, and DNAmAge acceleration were first described by boxplots and compared between tumor and normal tissues among each type of cancer patients and among cancer stage‐stratified patients by nonparametric Wilcoxon signed‐rank test. The performance of the three epigenetic markers for discriminating tumor tissues from normal tissues was evaluated using receiver operating characteristic (ROC) curves, and areas under the curve (AUCs) and confidence interval (CI) derived from logistic regression. All analyses were repeated in blood samples of CRC cases and controls.

Methylation data were preprocessed and normalized in r (version 3.2.3). All statistical analyses were conducted in sas 9.4 (SAS Institute, Cary, NC, USA).

## 3 Results

The analyses on paired tumor‐normal tissues were based on 612 cancer patients. Characteristics of those patients are presented in Table 1. The average age of each type of cancer patients (except for breast cancer [BC]) was above 60 years. Most patients had tumor diagnosed at stage I or stage II (except head‐and‐neck cancer).

**Table 1:** Characteristics of cancer patients.

Characteristics	LC (*n* = 69) TCGA	CRC		BC (*n* = 90) TCGA	Head‐and‐neck cancer (*n* = 50) TCGA	Prostate cancer (*n* = 50) TCGA	Liver cancer (*n* = 50) TCGA
		(*n* = 45) TCGA	(*n* = 254) DACHS				
Age (mean ± SD)	67.6 ± 11.0	69.4 ± 12.3	69.7 ± 10.5	57.9 ± 15.3	62.6 ± 10.7	62.4 ± 6.5	62.7 ± 16.1
Men (*N*/%)	43 (62.3)	24 (53.3)	124 (58.2)		38 (76.0)	50 (100.0)	30 (60.0)
Stages (*N*/%)							
I	40 (58.8)	5 (11.1)	41 (19.2)	13 (14.6)	–	–	21 (52.5)
II	11 (16.2)	21 (46.7)	79 (37.1)	55 (61.8)	8 (16.0)	–	8 (20.0)
III	14 (20.6)	10 (22.2)	65 (30.5)	20 (22.5)	10 (20.0)	–	10 (25.0)
IV	3 (4.4)	9 (20.0)	28 (13.2)	1 (1.1)	32 (64.0)	–	1 (2.5)

### 3.1 Mortality risk score (MRscore)

**Fig. 1:**
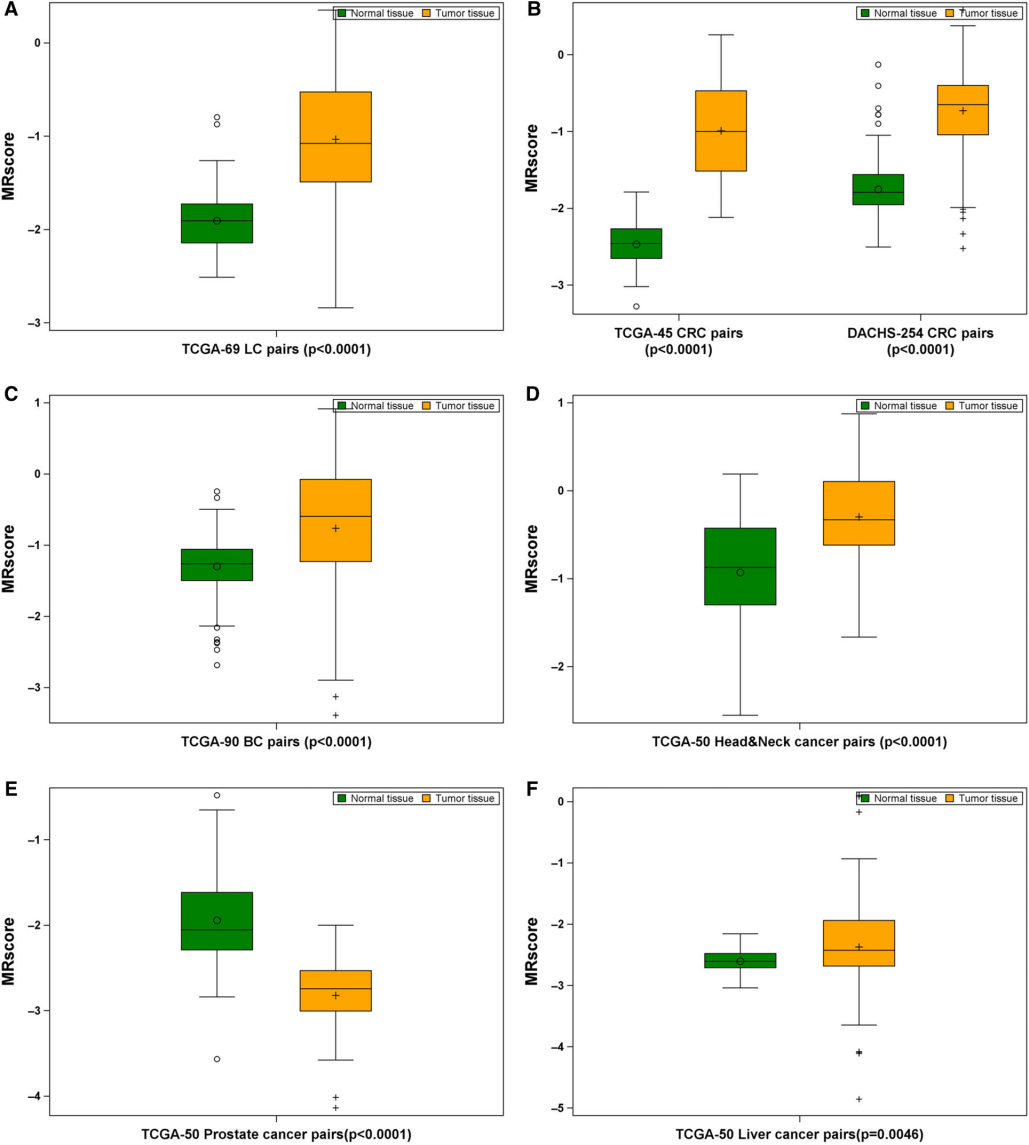
MRscore in tumor‐normal tissue pairs of patients with LC (*n* = 69; A), CRC (*n* = 299; B), BC (*n* = 90; C), head‐and‐neck cancer (*n* = 50; D), prostate cancer (*n* = 50; E), and liver cancer (*n* = 50; F). Difference between tumor and normal tissues was examined using nonparametric Wilcoxon signed‐rank test.

Figure 1 shows the levels of MRscore among tumor and normal tissues of all six types of cancer patients. Higher levels of MRscore in tumor tissue than in adjacent normal tissue were observed for five types of cancers (except prostate cancer), and the differences are most remarkable for CRC patients in both TCGA and the DACHS study (Fig. 1B). Consistently elevated MRscore in tumor tissues was also seen across stage I to stage IV tumors of each type of these cancers (Fig. S1). Among prostate cancer patients, tumor tissue showed significantly lower levels of the MRscore than normal tissues (Fig. 1E). This resulted from several CpGs that constitute the major components of the MRscore, which were hypermethylated in prostate tumor tissues (Fig. S2), such as cg06126421, cg24704287, and cg25983901, and have large absolute values of weights in the MRscore calculation, whereas these CpGs were hypomethylated in other tumor tissues, particularly for lung and CRC.

### 3.2 DNAmPhenoAge

**Fig. 2:**
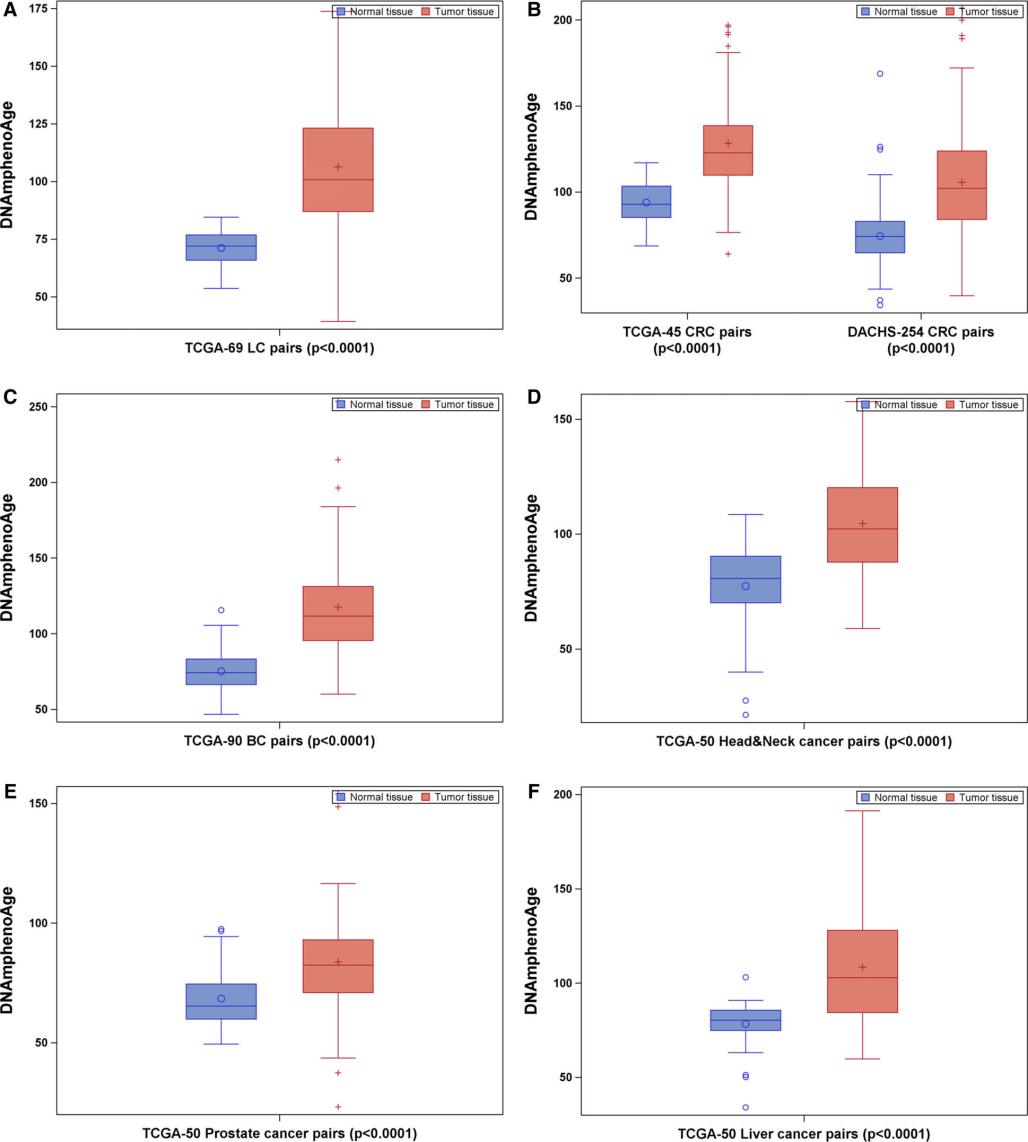
DNAmPhenoAge in tumor‐normal tissue pairs of patients with LC (*n* = 69; A), CRC (*n* = 299; B), BC (*n* = 90; C), head‐and‐neck cancer (*n* = 50; D), prostate cancer (*n* = 50; E), and liver cancer (*n* = 50; F). Difference between tumor and normal tissues was examined using nonparametric Wilcoxon signed‐rank test.

Tumor tissues of all six types of cancers exhibited significantly higher levels of DNAmPhenoAge than the corresponding adjacent normal tissues (Fig. 2). Consistent increases of DNAmPhenoAge across all stages of tumor tissues were also observed for all six types of cancers (Fig. S3).

### 3.3 DNAmAge acceleration

**Fig. 3:**
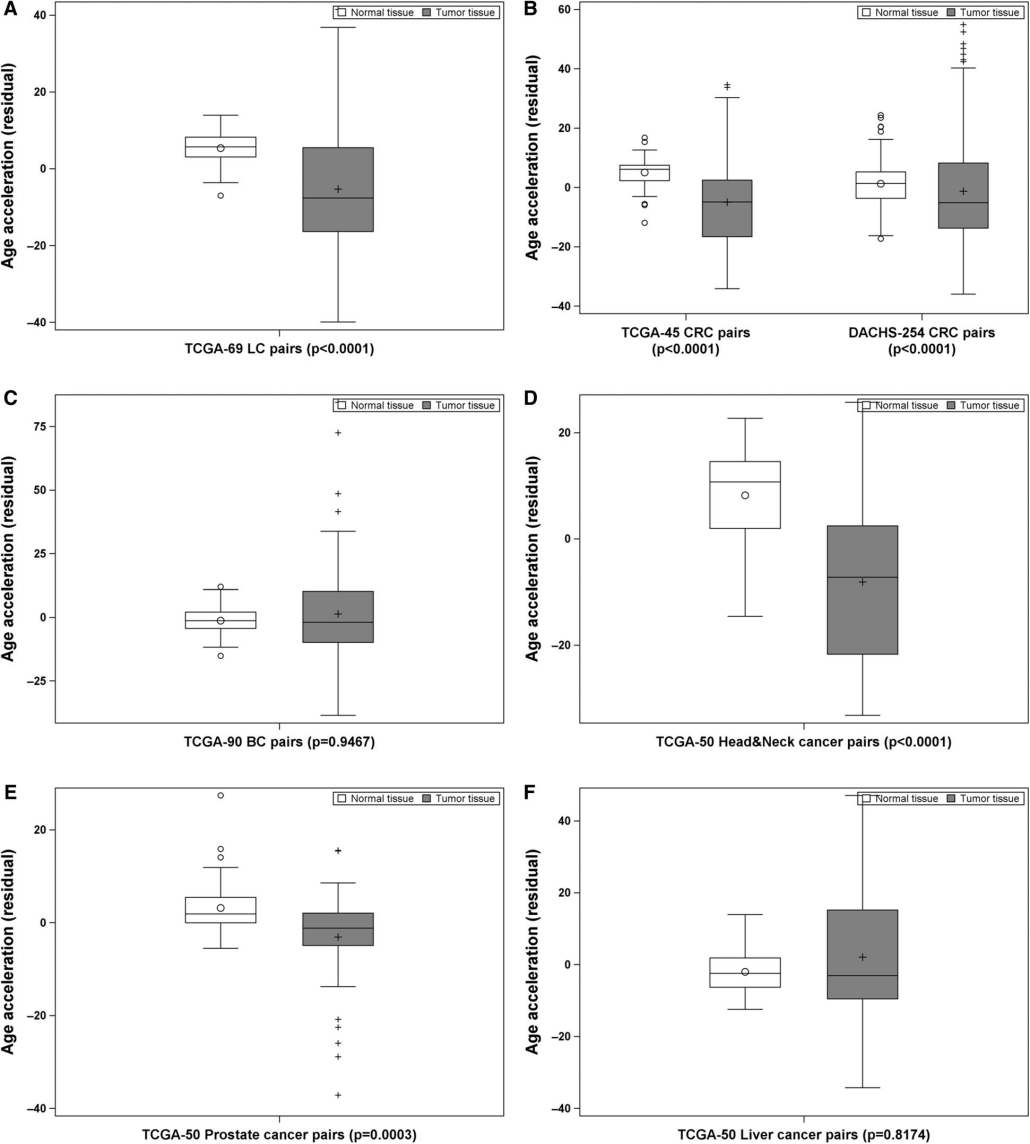
DNAmAge acceleration in tumor‐normal tissue pairs of patients with LC (*n* = 69; A), CRC (*n* = 299; B), BC (*n* = 90; C), head‐and‐neck cancer (*n* = 50; D), prostate cancer (*n* = 50; E), and liver cancer (*n* = 50; F). Difference between tumor and normal tissues was examined using nonparametric Wilcoxon signed‐rank test.

Unlike the pattern of the MRscore and DNAmPhenoAge, DNAmAge acceleration was lower among tumor tissues of lung, colorectal, head‐and‐neck, and prostate cancer patients, compared to the corresponding adjacent normal tissues (Fig. 3). No difference in DNAmAge acceleration between tumor and normal tissues of breast and liver cancer patients was seen (Fig. 3C,F). These patterns were also observed when stratifying patients by cancer stages (Fig. S4).

### 3.4 Discriminative performance of MRscore, DNAmPhenoAge, and DNAmAge acceleration

**Fig. 4:**
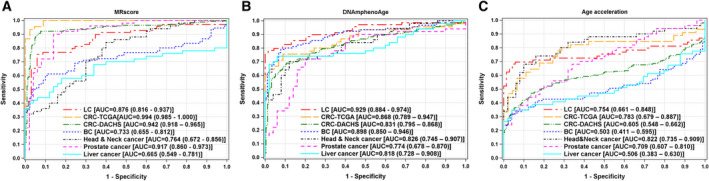
ROC curves for discriminating tumor tissues from normal tissues of common cancer patients by MRscore (A), DNAmPhenoAge (B), and DNAmAge acceleration (C).

Figure 4A shows that the MRscore can discriminate lung, colorectal, and prostate tumor tissues from normal tissues with very high accuracy (AUCs of 0.87–0.99). DNAmPhenoAge can accurately discriminate five of six types of tumor tissues (except prostate tumor) from normal tissues, with AUCs of 0.82–0.93 (Fig. 4B). Compared to the MRscore and DNAmPhenoAge, DNAmAge acceleration showed relatively lower and limited accuracy for discriminating five of six types of tumor tissues (except head‐and‐neck tumor) from normal tissues (Fig. 4C). Overall, the MRscore outperformed DNAmPhenoAge for colorectal and prostate cancer tissue discrimination, and DNAmPhenoAge outperformed the MRscore for breast and liver cancer tissue discrimination. Both MRscore and DNAmPhenoAge showed similarly high accuracy for lung cancer (LC) tissue discrimination.

### 3.5 MRscore, DNAmPhenoAge, and DNAmAge acceleration in blood samples of CRC cases and controls

**Fig. 5:**
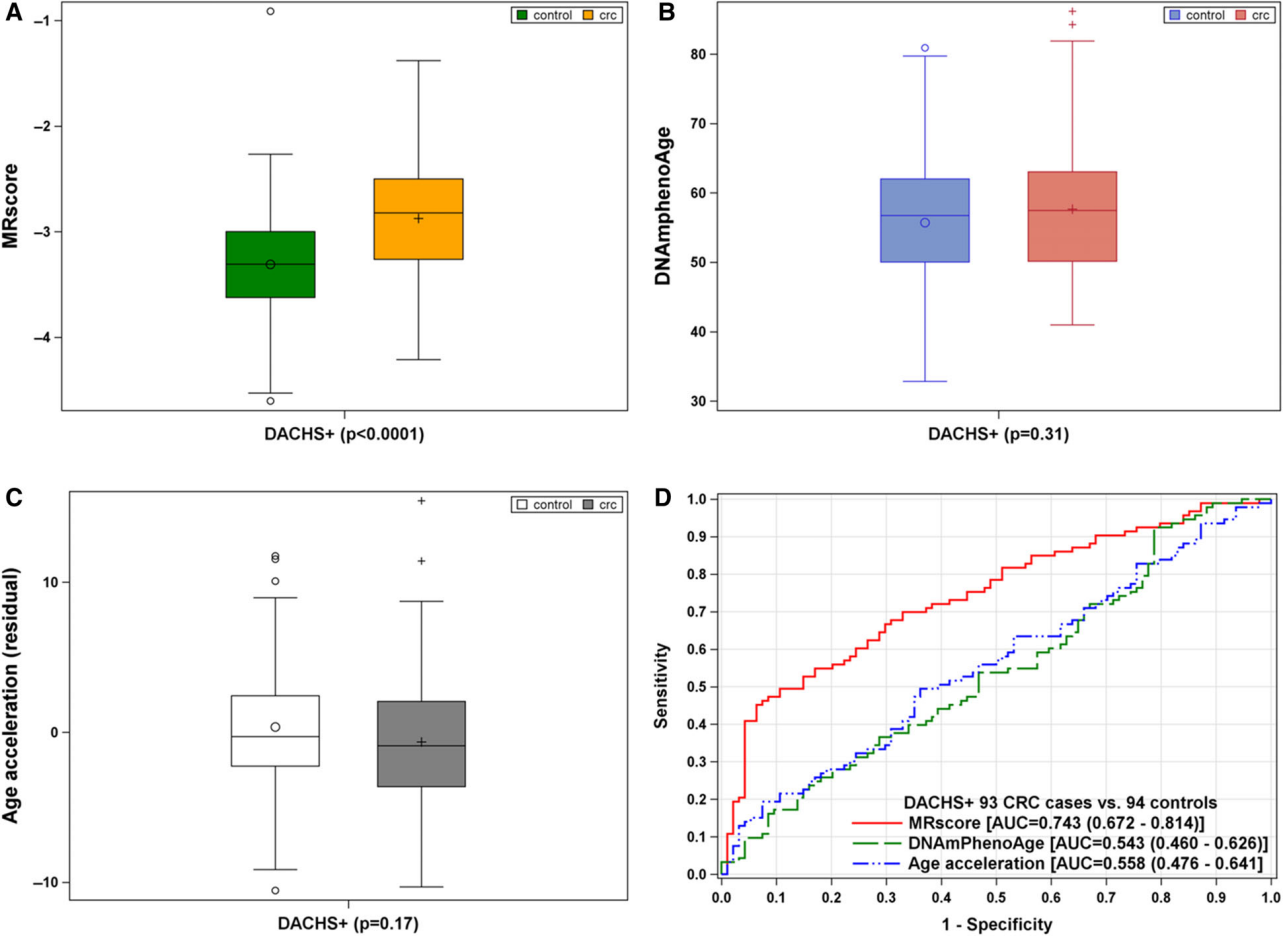
MRscore, DNAmPhenoAge, and DNAmAge acceleration in blood samples of CRC cases (*n* = 93) and controls (*n* = 94) [A‐C; difference between cases and controls was examined using nonparametric Wilcoxon signed‐rank test], and in discrimination of cases from controls in the DACHS+ study (D).

The characteristics of the CRC cases and controls are presented in Table S1. Distribution of major risk factors for CRC, such as age [mean (SD), 65 (8.4)], sex, smoking, body mass index, and colonoscopy history, is comparable between cases and controls. MRscore is significantly higher in cases than in controls (Fig. 5A), whereas no difference in both DNAmPhenoAge and DNAmAge acceleration was observed between cases and controls (Fig. 5B,C). The MRscore showed modest accuracy for discrimination of CRC cases from controls (AUC of 0.74) and outperformed DNAmPhenoAge and DNAmAge acceleration (AUCs of 0.54 and 0.56, respectively).

## 4 Discussion

Based on both publicly available and local DNAm data, the current study demonstrated distinct alterations of two strong survival predictors, that is, the MRscore and DNAmPhenoAge, in tumor tissue compared to adjacent normal tissue samples of patients with common forms of cancer. The MRscore accurately differentiated lung, colorectal, and prostate tumor tissues from normal tissues (AUCs of 0.87–0.99), and DNAmPhenoAge accurately differentiated lung, colorectal, breast, head/neck, and liver tumor tissues from adjacent normal tissues (AUCs of 0.82–0.93). Our findings thus further substantiate the evidence that the two predictors are robust epigenetic markers of health outcomes. The consistent pattern of MRscore across target tissue and blood samples of CRC patients strengthens the hypothesis that blood‐derived methylation markers could mirror tumorigenesis‐related methylation changes in less accessible tissues.

The MRscore was computed based on 10 CpGs derived from direct regression of mortality (time‐to‐death) on DNAm levels in a large EWAS, in which the majority of participants were healthy individuals at the time of blood collection and the MRscore strongly predicted their mortality during 14 years of follow‐up [12]. DNAm was quantified among participants with average age of 62 at baseline (range, 50–75 years), for whom common chronic diseases, such as malignant diseases, are the predominant causes of death [26]. The MRscore in nature thus captures and aggregates the risk of developing and surviving common chronic diseases including major cancers. The DNAmPhenoAge was developed through first estimating a phenotypic age based on clinical biomarkers and chronological age, followed by regressing this phenotypic age on DNAm levels of 513 CpGs [11]. This two‐step‐produced DNAmPhenoAge is thus in principle an estimator of biological aging, a major risk factor for cancer [27]. In line with the previous findings that high levels of MRscore and DNAmPhenoAge correlate with increased risk of adverse outcomes [11,12], we found elevated levels of the two markers in tumor tissues rather than in normal tissues. This suggests that regulation of DNAm patterns in CpGs included in the MRscore and DNAmPhenoAge could potentially be relevant for tumorigenesis in solid tissues.

Solid tumors are constituted of malignant cells as well as nonmalignant cell populations that largely overnumber tumor cells [28,29]. Tumors are thus abundantly infiltrated by leukocytes, so‐called tumor‐infiltrating leukocytes (TILs), which consist of immune cells (such as T cells, B cells, natural killer cells, macrophages, neutrophils, and eosinophils) and play a fundamental role in cancer immune surveillance [30]. Consistent methylation changes in the TILs and leukocytes in the peripheral blood might explain why the MRscore and DNAmPhenoAge, which were originally derived from blood sample analysis, showed such strong discrimination between tumor and adjacent tissues for most of the cancers studied. In the current study, we also observed variations of the performance of the MRscore and DNAmPhenoAge in tumor/normal tissue discrimination between different types of tumors, that is, the MRscore outperformed DNAmPhenoAge for colorectal and prostate cancer tissue discrimination, whereas DNAmPhenoAge outperformed the MRscore for breast and liver cancer tissue discrimination. This, on the one hand, may result from differential methylation profiles of the two markers, with a complete lack of overlap between the 10 CpGs in the MRscore and the 513 CpGs in DNAmPhenoAge. On the other hand, the differences may also be explained by heterogeneity of the TILs across various tumors, as compelling evidence from tumor immunology has shown that the abundance and composition of TILs strikingly vary with tumor type and indicate differential prognostic and predictive value [28, 29, 30]. Furthermore, pathophysiological, immune infiltrate promoted inflammation in tumor microenvironment is consistent with potential biological functions of the DNAm markers in the MRscore and DNAmPhenoAge. For example, in addition to four CpGs in the MRscore mapped to genes involved in various cancers (cg23665802 in *MIR19A,* cg08362785 in *MKL1,* cg19572487 in *RARA,* cg05575921 in *AHRR*) [31, 32, 33, 34, 35, 36, 37, 38, 39], three CpGs in the MRscore (cg05575921, cg06126421, and cg08362785) were identified to correlate with C‐reactive protein, a sensitive indicator of chronic inflammation, in a meta‐analysis of EWAS [40]. Six CpGs in the MRscore are strongly related to tobacco smoking [12,41,42], a factor with well‐established strong effects on inflammation/immune processes [43,44] and 18 types of cancer such as lung, colorectal, and liver cancer [45], [46]. For DNAmPhenoAge, Levine *et al*. [11] conducted GO enrichment analysis and observed enrichment for a number of pro‐inflammatory signaling pathways, including but not limited to regulation of inflammatory response, tumor necrosis factor‐mediated signaling pathway, and positive regulation of NFkappaB transcription factor activity. Taken together, notwithstanding the need to further unravel a clear picture of how the MRscore and DNAmPhenoAge are related to cancer development and progression, TILs and their involved inflammatory/immunologic responses may explain our findings.

Previous EWASs that investigated cancer‐related DNAm signatures across the whole genome through direct comparison of tumor and adjacent normal tissues have disclosed numerous differentially methylated CpG sites of tumor tissues relative to normal tissues [22,47,48]. It is thus not difficult to derive algorithms with sufficient amount of CpGs, which could reach high distinction between tumor and normal tissues when analyzing tissue samples. However, algorithms derived in such a way usually exhibit completely different performance in nontarget tissues and can hardly be transferred to blood samples for clinical application, whereas the MRscore and DNAmPhenoAge bear potential concordance in blood and target solid tissues, as illustrated by the MRscore that elevated in both tumor tissue and blood samples of CRC patients in the current study. In addition, we in parallel assessed the well‐studied Horvath's epigenetic clock, which was derived from multitissue analysis and combined far more CpGs (*n* = 353) than the MRscore (*n* = 10). Our study did not yield evidence that this epigenetic clock outperformed the MRscore in terms of tumor‐normal tissue discrimination, even though it has been linked to various chronic diseases including cancers, such as LC and BC [18, 19, 20]. This finding is also not surprising given that the effect sizes for the associations of the epigenetic clock reflected age acceleration with health conditions are typically small or moderate. Nevertheless, it is worthwhile pointing out that the MRscore and DNAmPhenoAge are indicative of the presence of multiple cancers rather than of a specific cancer. However, variations of the MRscore and DNAmPhenoAge between cancers were also found, including but not limited to the opposite pattern of MRscore in prostate cancer tissue vs. normal tissue; thus, their applications in specific cancers need to be explored by future research.

In contrast to the MRscore and DNAmPhenoAge that were developed to capture the risk and physiological dysregulation [11,12], Horvath's epigenetic clock was built upon methylation markers that are strongly correlated with chronological age and trained to be an age estimator [10]. In the current study, we found, compared to the consistent pattern of the MRscore and DNAmPhenoAge in tumor vs. normal tissues, opposite patterns for the DNAmAge acceleration based on Horvath’s epigenetic clock. This is in line with findings from previous studies that in many types of cancer tissues, age‐associated DNAm signatures hardly correlated with chronological age of the cancer patients and DNAmAge is often predicted to be younger [49,50]. A plausible explanation is that DNAmAge estimated in tumor tissue may partly reflect the state of aging in the tumor‐initiating cells, the cancer stem cells, which exhibit young biological age [10]. However, a caveat needs to be considered given the relatively small sample size of each cancer type in the current study.

In the current study, although we examined the DNAm‐based algorithms in several common forms of cancer and yielded basically consistent patterns across most cancers, the sample size for each cancer type was rather limited (< 100 pairs of tissue samples except for CRC). Another major limitation of the current study is that we assessed the DNAm‐based algorithms in paired tissues and blood samples only for CRC patients, and were not able to investigate their levels in both target tissues and blood samples of other cancer patients and healthy controls. The analyses for CRC were not conducted in tissues and blood samples from the same patients, which may bring up additional variation. Disconcordance of DNAmPhenoAge between tissue and blood samples based on the current CRC analyses thus should be interpreted with caution and should not be extrapolated to other types of cancers. Future studies with a large number of ‘tripled samples’, including blood, tumor, and adjacent normal tissues of the same patients, along with blood samples from tumor‐free participants, are needed to confirm our findings, clarify the relevant biological pathways, and evaluate the potential use of the blood‐based algorithms for cancer diagnosis and early detection.

## 5 Conclusions

The current study demonstrated that two DNAm‐based algorithms, which were previously shown to be strongly predictive of mortality when measured in blood samples, were also indicative of methylation changes in tissues of various common cancers, suggesting that they might reflect tumor‐related methylation changes. TILs and the underlying inflammatory process may explain potential concordance of methylation changes in both blood and solid tumor tissues, which, however, needs to be further explored by future studies with simultaneous collection and analyses of blood and tissue samples. Given the properties of easy accessibility and processing of whole blood samples, the use of DNAm‐based algorithms for cancer diagnosis and early detection should be explored in future research.

## Conflict of interest

The authors declare no conflict of interest.

## Author contributions

YZ and HB designed and conceived the study. YZ retrieved the data, carried out the analyses, and drafted the manuscript. HB, JC, and MH are the principal investigators of the DACHS study and carried out, led, and supervised its conduction. MS, MB, and BB contributed to processing the DACHS DNA with Illumina Human Methylation arrays. EH contributed to the provision of DACHS tissue samples. All authors contributed to the revision of the manuscript and approved the final version for submission.

## Supporting information


**Fig**.** S1**. Stage‐specific levels of MRscore in paired tumor and normal tissues of 69 lung cancer (LC) patients from the TCGA (A), 45 colorectal cancer (CRC) patients from the TCGA (B), 254 CRC patients from the DACHS study (C), 90 breast cancer (BC) patients from the TCGA (D), 50 head‐and‐neck (HN) cancer patients from the TCGA (E), and 50 liver cancer patients from the TCGA (F).
**Fig**.** S2**. Methylation level of the 10 CpGs in MRscore by tumor and normal tissues of LC (A), CRC (B), BC (C), head/neck cancer (D), prostate cancer (E), and liver cancer (F) patients from the TCGA, and of CRC (G) patients from the DACHS study.
**Fig**.** S3**. Stage‐specific levels of DNAmPhenoAge in paired tumor and normal tissues of 69 LC patients from the TCGA (A), 45 CRC patients from the TCGA (B), 254 CRC from the DACHS study (C), 90 BC patients from the TCGA (D), 50 HN cancer patients from the TCGA (E), and 50 liver cancer patients from the TCGA (F).
**Fig**.** S4**. Stage‐specific levels of DNAmAge acceleration in paired tumor and normal tissues of 69 LC patients from the TCGA (A), 45 CRC patients from the TCGA (B), 254 CRC from the DACHS study (C), 90 BC patients from the TCGA (D), 50 HN cancer patients from the TCGA (E), and 50 liver cancer patients from the TCGA (F).
**Table S1**. Characteristics of the CRC cases and controls in the DACHS+ study.

## Data Availability

TCGA DNAm and clinical data are available in the Cancer Genome Atlas database (https://www.cancer.gov/about‐nci/organization/ccg/research/structural‐genomics/tcga). For the DACHS study, due to restrictions of informed consent we are not allowed making the dataset publicly available. However, the use of the data for collaboration projects has been and will remain the approach for data sharing.
