# Supplementary material for: Blood‐derived DNA methylation predictors of mortality discriminate tumor and healthy tissue in multiple organs
Source: Mol Oncol. 2020 Jun 22;14(9):2111–23. doi: 10.1002/1878-0261.12738 (PMC7463320; doi:10.1002/1878-0261.12738)
Supplement: Supplementary file 1 — Fig. S1. Stage‐specific levels of MRscore in paired tumor and normal tissues of 69 lung cancer (LC) patients from the TCGA (A), 45 colorectal cancer (CRC) patients from the TCGA (B), 254 CRC patients from the DACHS study (C), 90 breast cancer (BC) patients from the TCGA (D), 50 head‐and‐neck (HN) cancer patients from the TCGA (E), and 50 liver cancer patients from the TCGA (F). Fig. S2. Methylation level of the 10 CpGs in MRscore by tumor and normal tissues of LC (A), CRC (B), BC (C), head/neck cancer (D), prostate cancer (E), and liver cancer (F) patients from the TCGA, and of CRC (G) patients from the DACHS study. Fig. S3. Stage‐specific levels of DNAmPhenoAge in paired tumor and normal tissues of 69 LC patients from the TCGA (A), 45 CRC patients from the TCGA (B), 254 CRC from the DACHS study (C), 90 BC patients from the TCGA (D), 50 HN cancer patients from the TCGA (E), and 50 liver cancer patients from the TCGA (F). Fig. S4. Stage‐specific levels of DNAmAge acceleration in paired tumor and normal tissues of 69 LC patients from the TCGA (A), 45 CRC patients from the TCGA (B), 254 CRC from the DACHS study (C), 90 BC patients from the TCGA (D), 50 HN cancer patients from the TCGA (E), and 50 liver cancer patients from the TCGA (F). Table S1. Characteristics of the CRC cases and controls in the DACHS+ study. [file MOL2-14-2111-s001.docx]

**Supplementary Appendix to**

**Blood-derived DNA methylation predictors of mortality discriminate tumor and healthy tissue in multiple organs**

**Figure S1.** Stage-specific levels of MRscore in paired tumor and normal tissues of 69 lung cancer (LC) patients from the TCGA (A), 45 colorectal cancer (CRC) patients from the TCGA (B), 254 CRC patients from the DACHS study (C), 90 breast cancer (BC) patients from the TCGA (D), 50 head-and-neck (HN) cancer patients from the TCGA (E), and 50 liver cancer patients from the TCGA (F).

**Figure S2**. Methylation level of the 10 CpGs in MRscore by tumor and normal tissues of LC (A), CRC (B), BC (C), head/neck cancer (D), prostate cancer (E), and liver cancer (F) patients from the TCGA, and of CRC (G) patients from the DACHS study.

**Figure S3.** Stage-specific levels of DNAmphenoAge in paired tumor and normal tissues of 69 LC patients from the TCGA (A), 45 CRC patients from the TCGA (B), 254 CRC from the DACHS study (C), 90 BC patients from the TCGA (D), 50 HN cancer patients from the TCGA (E), and 50 liver cancer patients from the TCGA (F).

**Figure S4.** Stage-specific levels of DNAmAge acceleration in paired tumor and normal tissues of 69 LC patients from the TCGA (A), 45 CRC patients from the TCGA (B), 254 CRC from the DACHS study (C), 90 BC patients from the TCGA (D), 50 HN cancer patients from the TCGA (E), and 50 liver cancer patients from the TCGA (F).

**Table S1.** Characteristics of the CRC cases and controls.

Figure S1. Stage-specific levels of MRscore in paired tumor and normal tissues


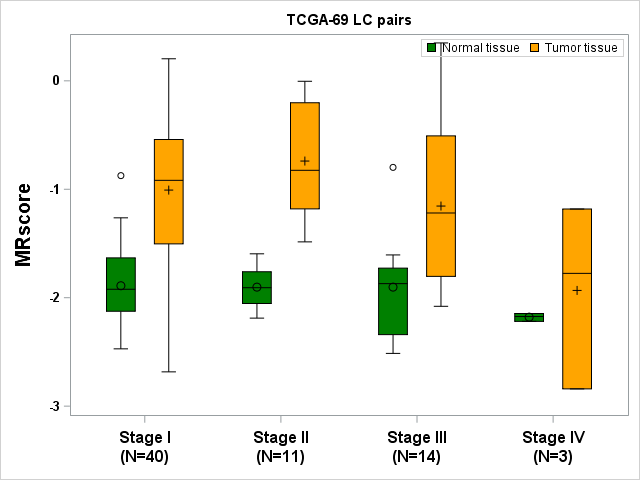

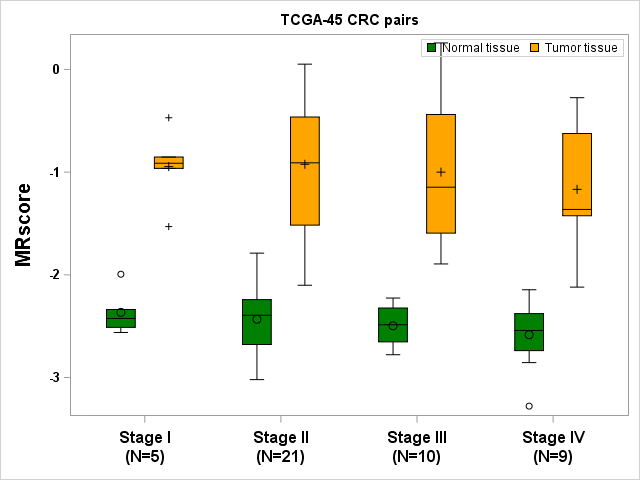

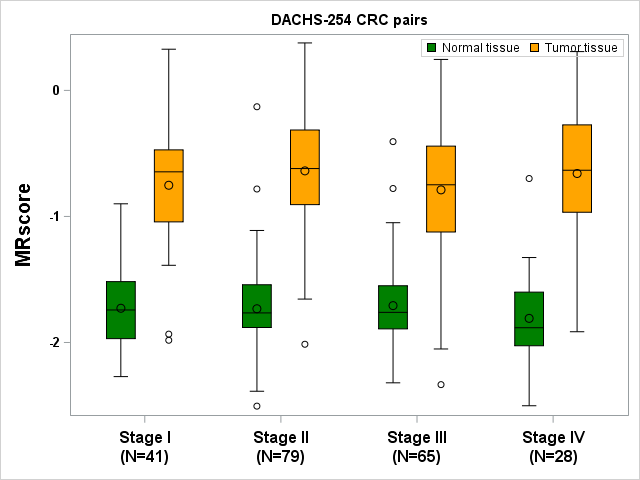

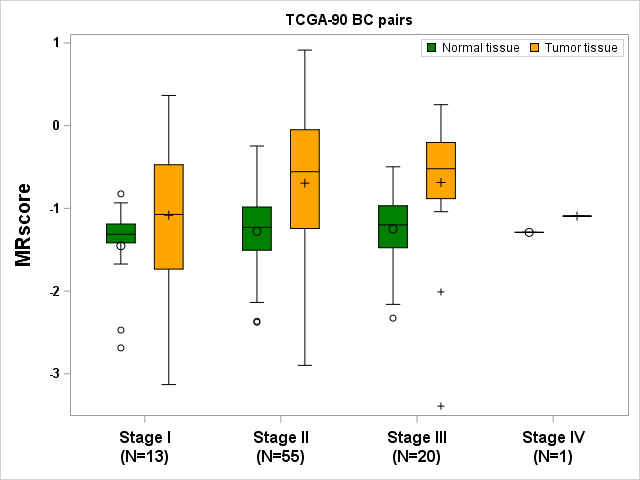

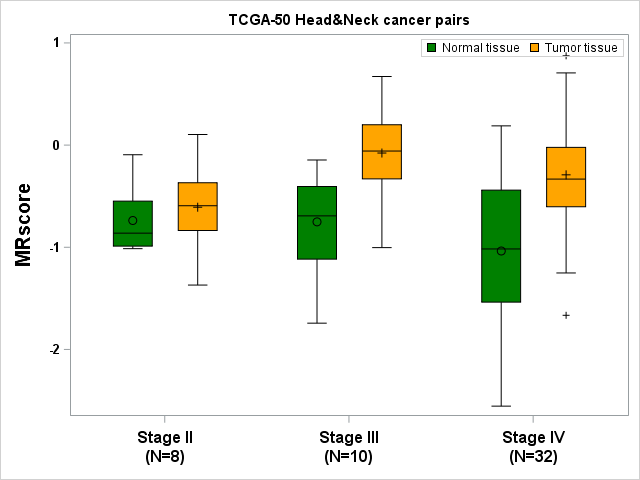

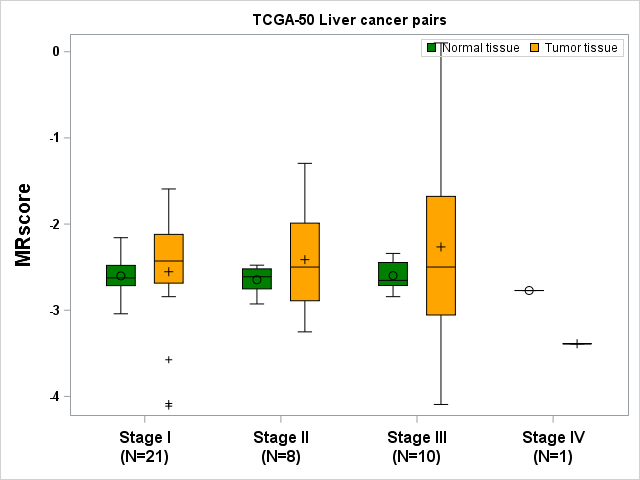


**F.**

**E.**

**D.**

**C.**

**B.**

**A.**

Figure S2. Methylation level of the 10 CpGs in MRscore by tumor and normal tissues


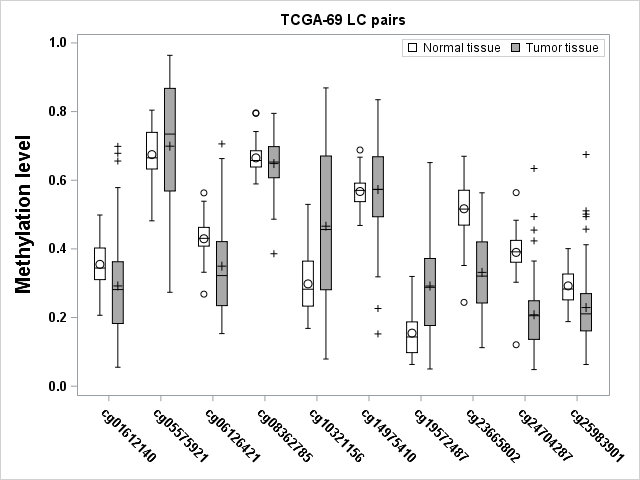

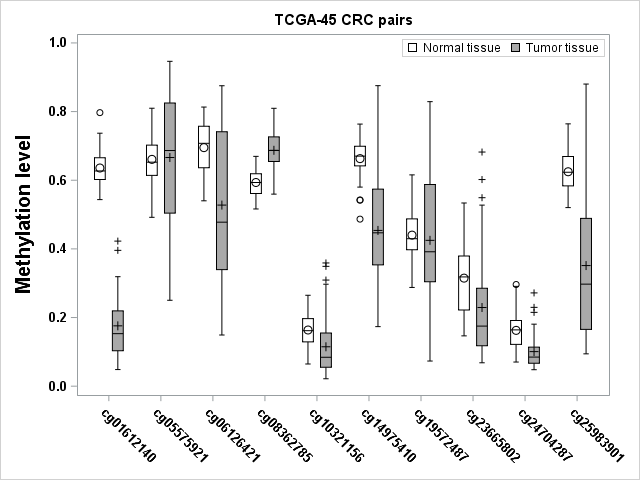

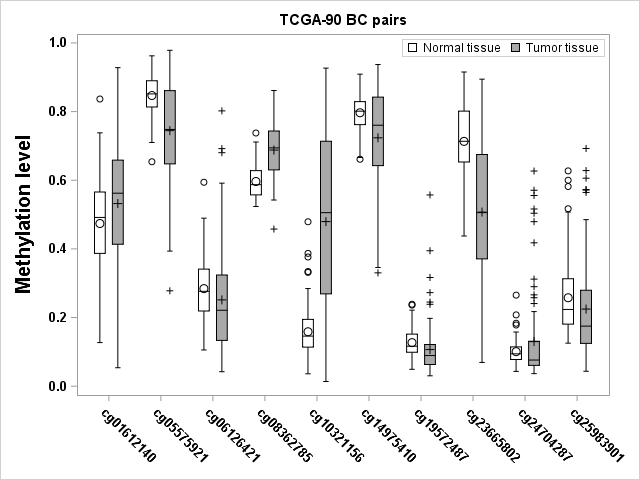

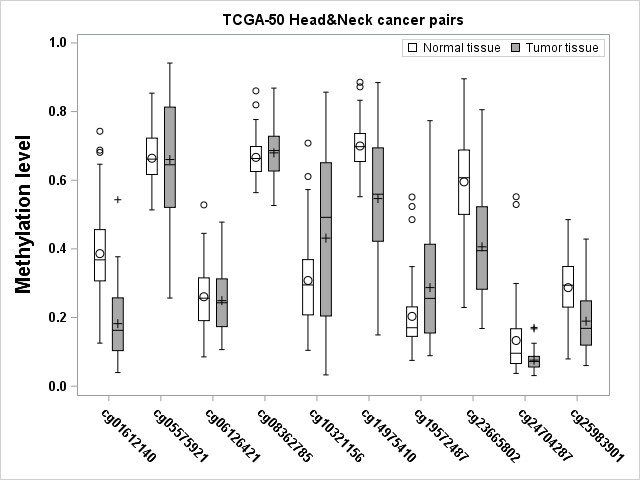

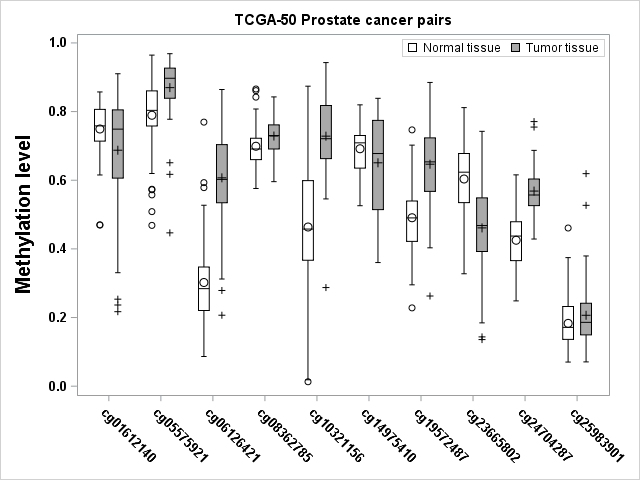

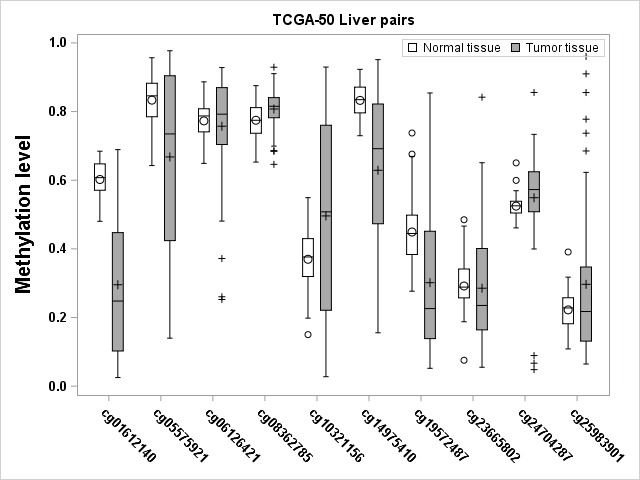


**F.**

**E.**

**D.**

**C.**

**B.**

**A.**


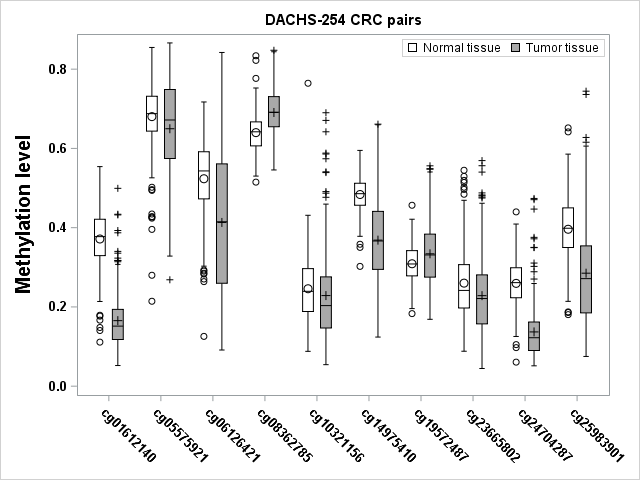


**G.**

Figure S3. Stage-specific levels of DNAmphenoAge in paired tumor and normal tissues


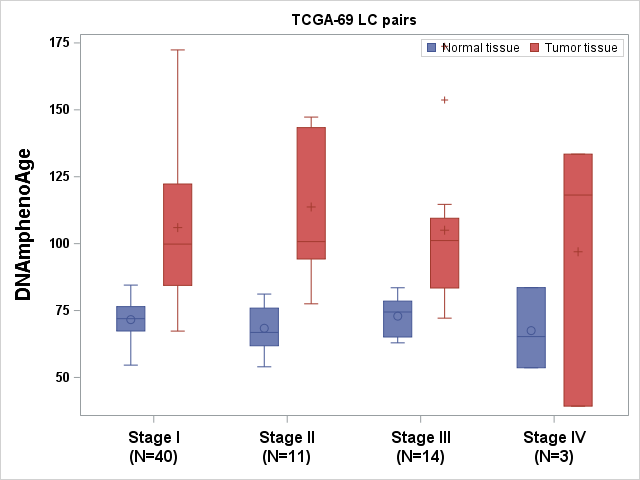

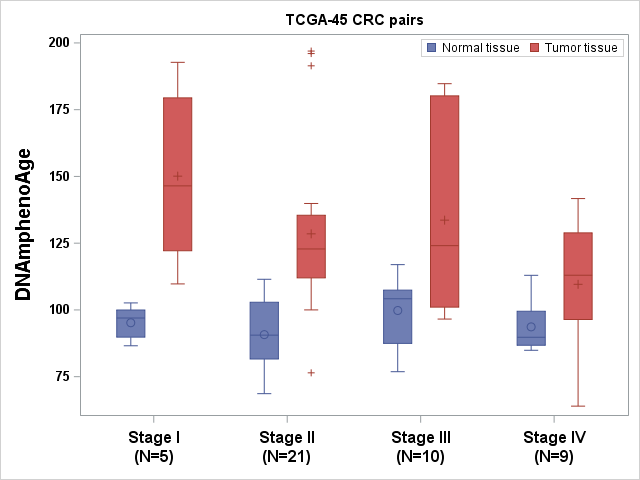


**B.**

**A.**


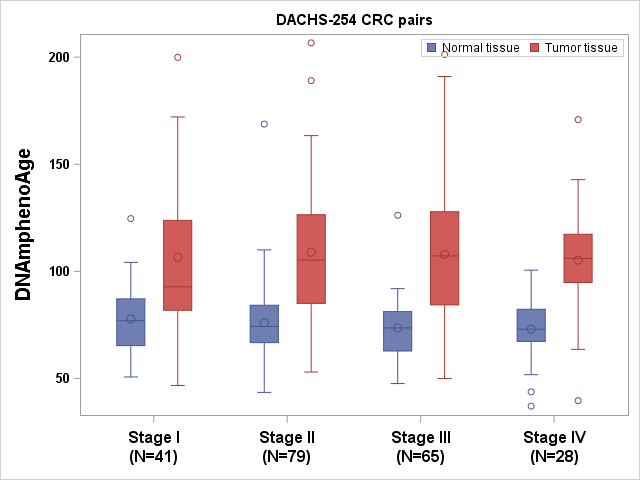

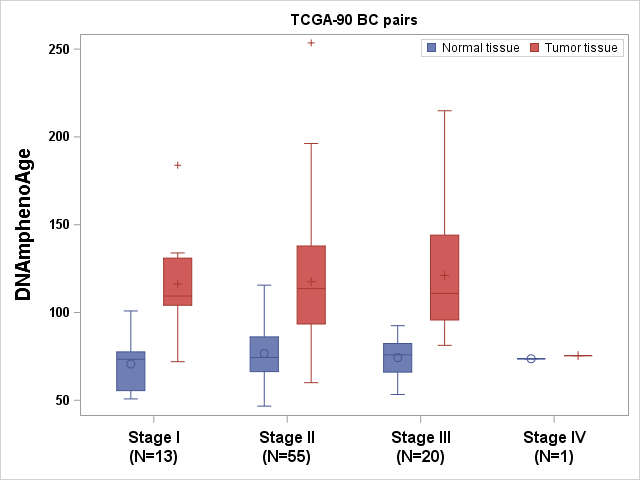

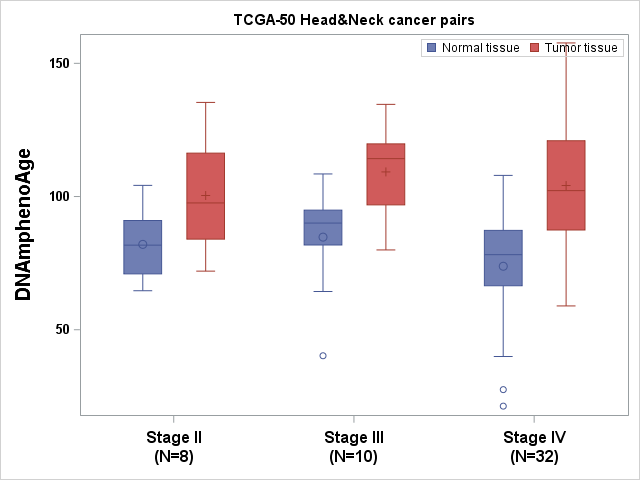

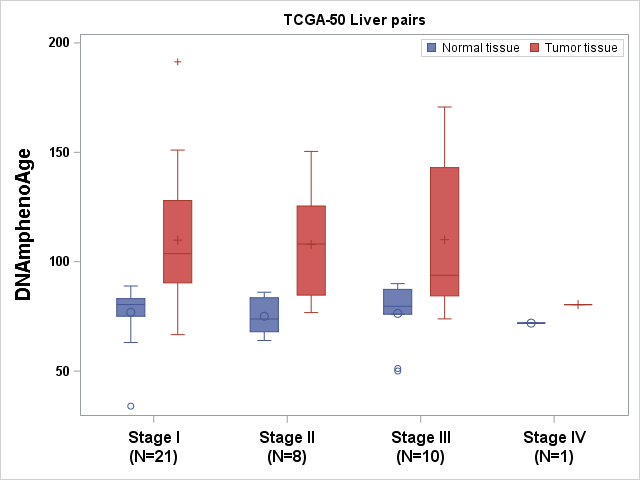


**F.**

**E.**

**D.**

**C.**

**B.**

**D.**

Figure S4. Stage-specific levels of DNAmAge acceleration in paired tumor and normal tissues


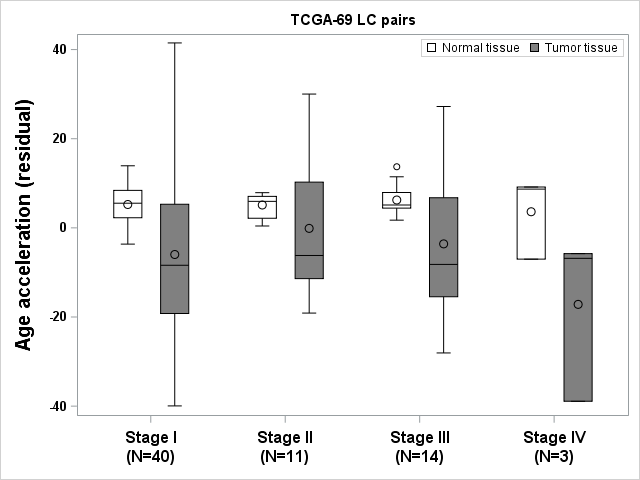

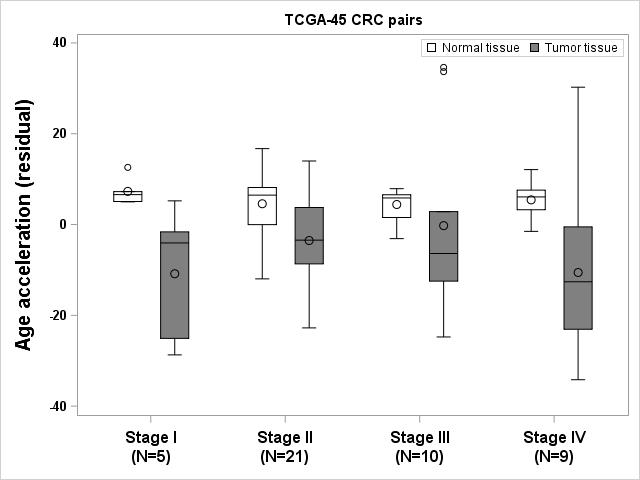

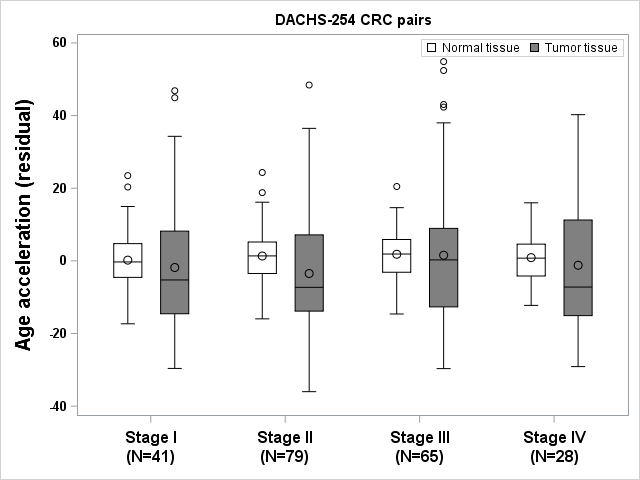

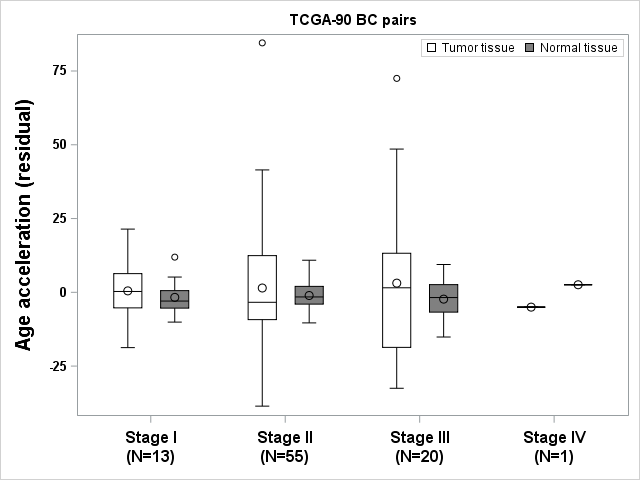

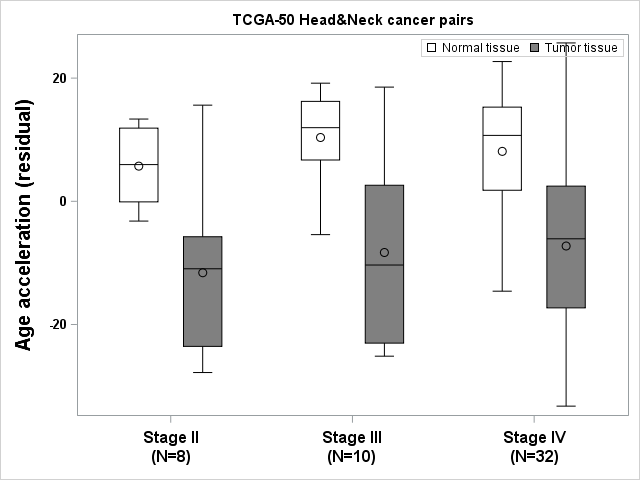

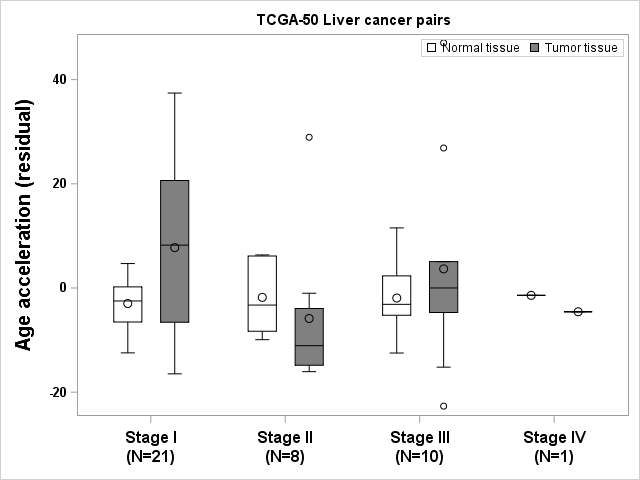


**Table S1.** Characteristics of the CRC cases and controls in the DACHS+ study

| Characteristics | CRC cases  (n=93) | Controls  (n=94) | P-value |
| --- | --- | --- | --- |
| Age (mean ± SD) | 65.1 ± 8.4 | 65.3 ± 8.4 | 0.95 |
| Men (N/%) | 57 (61.3) | 57 (60.6) | 0.93 |
| Ever smoker (N/%) | 46 (52.9) | 47 (50.0) | 0.70 |
| Alcohol consumption, g/day (mean ± SD) | 14.6 ± 25.8 | 8.9 ± 11.4 | 0.89 |
| BMI, kg/m^2^ (mean ± SD) | 26.9 ± 4.5 | 27.1 ± 4.2 | 0.54 |
| Family history of CRC (N/%) | 36 (38.7) | 13 (13.8) | 0.0001 |
| NSAIDs use (N/%) | 16 (17.2) | 22 (23.4) | 0.29 |
| Previous colonoscopy (N/%) | 29 (31.2) | 31 (33.0) | 0.33 |

Abbreviations: BMI, body mass index; CRC, colorectal cancer; NSAID, Nonsteroidal anti-inflammatory drugs; SD, standard deviation.

^a^P-value for testing difference between overall cases and controls by Wilcoxon signed-rank test for continuous variables and by chi-squared test for categorical variables.
